# Deployment of a machine learning-based predictive system for childhood diarrhea in Sub-Saharan Africa

**DOI:** 10.1038/s41598-026-43140-4

**Published:** 2026-03-13

**Authors:** Eliyas Addisu Taye, Eyob Akalewold Alemu, Halima Ayalew Kebede, Helen Brhan Alemaw, Simachew Getaneh Endalamew, Sofiya Ayalew Kebede, Solomon Keflie Assefa, Adem Tsegaw Zegeye, Belayneh Jejaw Abate, Dejen Kahsay Asgedom, Endalew Minwuye Andargie

**Affiliations:** 1 Department of Health Informatics, College of Medicine and Health Science, University of Gondar Comprehensive Specialized Hospital, Gondar, Ethiopia; 2 Department of Epidemiology and Biostatistics, Institute of Public Health, College of Medicine and Health Science, University of Gondar, Gondar, Ethiopia; 3 Department of Public Health, College of Medicine and Health Science, Woldia University, Woldia, Ethiopia; 4 Department of Veterinary Epidemiology and Public Health, School of Veterinary Medicine, Bahir Dar University, Bahir Dar, Ethiopia; 5 Department of Epidemiology and Biostatistics, School of Public Health, College of Medicine and Health Sciences, Wollo University, Dessie, Ethiopia; 6 Department of Public Health, School of Public Health, Debre Berhan University, Asrat Woldeyes Health Science Campus, Debre Berhan, Ethiopia; 7 University of Gondar Comprehensive Specialized Hospital, Gondar, Ethiopia; 8 Department of Public Health, College of Medicine and Health Science, Samara University, Samara, Ethiopia

**Keywords:** Machine learning, Diarrhea prediction, Child health, Sub-Saharan Africa, Computational biology and bioinformatics, Health care, Mathematics and computing, Medical research

## Abstract

Diarrhea remains a leading cause of child mortality in Sub-Saharan Africa, necessitating advanced predictive tools for early intervention. Despite the growing adoption of machine learning in healthcare, gaps persist in deploying models as scalable, real-world solutions. This study developed an end-to-end machine learning framework to predict diarrhea among children under five in SSA, integrating rigorous model development with Flask-based deployment for practical use. Using nationally representative Demographic and Health Surveys (DHS) data from 27 SSA countries (2016-2024), we preprocessed data (handling missing values, feature selection, and SMOTE for class imbalance), trained a Random Forest classifier (optimized via RandomizedSearchCV), and deployed the model as a RESTful API with Flask. The final model demonstrated strong predictive power, with 79.6% accuracy and a particularly high recall of 84.1%, meaning it is exceptionally effective at identifying true diarrhea cases. Most importantly, the model is no longer just a research output; it is a deployed, interactive system ready for practical application. This work successfully demonstrates a complete pipeline from data to deployment, offering a tangible solution that can aid public health decision-making. We have proven that it is possible to close the gap between machine learning research and real-world implementation. To build on this foundation, future work should focus on enhancing the model’s interpretability for health workers, adopting more scalable deployment technologies like FastAPI and Docker, and conducting rigorous field validation with community stakeholders to ensure these tools truly meet the needs of those they are designed to serve.

## Introduction

 The landscape of modern technology has increasingly incorporated Machine Learning (ML), a field that holds considerable potential across diverse sectors, including healthcare diagnostics, financial fraud detection, e-commerce recommendations, and autonomous systems^1–5^. While ML models are capable of identifying complex patterns within large datasets and supporting informed predictions or decisions, their translation into impactful, real-world applications, particularly in healthcare remains limited and often challenging. The true value of an ML model therefore extends beyond theoretical performance; it relies on careful documentation of the research and development process, as well as practical operationalization through robust deployment mechanisms^6–8^.

This report aims to bridge the gap between academic rigor and practical application by detailing a formal approach to ML research, emphasizing the complete pipeline from data analysis to deployment. To establish a common understanding, foundational ML concepts are first introduced. In ML dataset consists of data points, also referred to as samples, represent individual entities within a dataset. Each data point is characterized by features, which are attributes that define its position within a multi-dimensional feature space. The values of all features for a given data point collectively form a feature vector. Machine learning primarily operates within two main domains: supervised learning, where models learn to predict a class or value based on pre-existing labeled data, and unsupervised learning, where models identify unknown patterns in data without explicit labels^9,10^.

The structured documentation of machine learning research, as advocated by academic guidelines, is not merely a formality; it is fundamental to ensuring the reproducibility of an ML model’s development and evaluation. When this structured documentation is combined with detailed, and executable deployment instructions, it creates a complete, verifiable lifecycle of an ML solution. This integration accelerates scientific progress and practical application, moving machine learning from purely theoretical exploration to tangible, usable solutions. The emphasis on providing publishable deployment docummenation directly addresses the critical need for practical reproducibility, allowing other researchers and practitioners to not only understand what was achieved but also how it was implemented and, crucially, how to utilize it in real-world scenarios^11,12^.

The core problem addressed by the underlying machine learning project, which this report formally documents, revolves around the challenge of translating advanced analytical models into accessible, real-world applications^1,13^. Predictive models often remain confined to experimental environments, such as Jupyter notebooks, significantly limiting their real-world impact. Deployment serves as the crucial bridge, enabling user interaction via web interfaces and thus unlocking the full potential of these models^14^. Existing knowledge provides extensive methodologies for model training and evaluation. However, the formal articulation and standardization of the deployment phase, particularly for lightweight, accessible solutions, remain less emphasized in traditional research documentation^14^.

This study addresses a critical gap in public health by focusing on diarrhea prediction among children under five in Sub-Saharan Africa (SSA), a region burdened by high child mortality rates linked to preventable diseases. By leveraging nationally representative Demographic and Health Surveys (DHS) data from 27 countries, the research not only advances methodological rigor in machine learning but also demonstrates tangible applications for improving healthcare outcomes. The deployed Flask-based API ensures that the model’s predictive capabilities are accessible to healthcare providers and policymakers, enabling timely interventions. This work underscores the transformative potential of integrating machine learning with scalable deployment frameworks to address pressing global health challenges.

## Methodology

### Study design and study period

This study utilized data from the DHS conducted across 27 SSA countries between 2016 and 2024 that had been collected through a cross-sectional survey design. The DHS datasets are nationally representative and provide comprehensive information on health, fertility, and demographic characteristics, collected through surveys designed to capture a broad spectrum of maternal and child health issues. The data used in this analysis is publicly available and can be accessed from the DHS program’s official website at https://dhsprogram.com/data/available-datasets.cfm. This study was conducted across 27 countries in SSA, a region that comprises nations located geographically south of the Sahara Desert. SSA encompasses four major regions: Central Africa, East Africa, Southern Africa, and West Africa^15^. According to The World Academy of Sciences, the region consists of 50 countries^16^, and it has experienced rapid demographic changes over the decades. The population in SSA grew from 186 million in 1950 to 856 million in 2010^17^. In 2023, the total population of SSA amounted to approximately 1.26 billion inhabitants^18^.

### Data sources

For this study, DHS datasets from selected Sub-Saharan African countries with recent survey data (2016-2024) were used, including Angola (4.7%), Burkina Faso (4.1%), Benin (4.4%), Burundi (4.3%), Cameroon (3.1%), Côte d’Ivoire (3.4%), Ethiopia (3.5%), Gabon (2.1%), Ghana (3.1%), Gambia (2.7%), Guinea (2.5%), Kenya (6.5%), Liberia (1.8%), Lesotho (0.8%), Madagascar (4.1%), Malawi (5.7%), Mali (3.2%), Mauritania (3.9%), Mozambique (3.1%), Nigeria (10.6%), Rwanda (2.7%), Senegal (3.5%), Sierra Leone (3.1%), South Africa (1.2%), Tanzania (3.6%), Uganda (5.1%), and Zambia (3.3%). The source population comprised all children under five years of age (≤ 59 months) residing in these countries. The study population specifically included children recorded in the Birth Recode (BR) datasets of the DHS surveys conducted between 2016 and 2024. After applying inclusion criteria, a total of 289,720 children were included in the final analysis.

### Source and study population

The source population for this study consisted of all children under five years of age (≤ 59 months) residing in SSA. The study population specifically included children within this age range who were recorded in the Birth Recode (BR) datasets of the DHS conducted between 2016 and 2024 across selected SSA countries.

### Study variables

The outcome variable in this study was diarrhea, measured as a binary variable (yes/no). This was assessed based on the mother’s response to the standardized DHS question: “Has your child had diarrhea in the last two weeks?” Children whose mothers reported that they had experienced diarrhea within the two weeks preceding the survey were classified as having diarrhea (coded as ‘1’), while those who had not were classified as not having diarrhea (coded as ‘0’). This self-reported measure provides a proxy indicator for recent episodes of diarrheal illness among children under five years of age^19^. A thorough review of the literature and DHS documentation was conducted to guide the selection of independent variables relevant to predicting diarrhea among children under the age of five. The independent variables considered for predicting diarrhea among under-five children included a wide range of maternal, child, household, and healthcare-related factors. These variables were: age of mothers, place of residence, highest educational level, wealth index, maternal literacy, smoking status (smokes cigarettes), husband’s education level, current employment status of the respondent, number of living children, parity, age of the respondent at first birth, whether the last child was wanted, fertility preference, challenges in getting permission to seek medical help, difficulties in obtaining money for treatment, distance to health facilities, and reluctance to go alone to health facilities. Additionally, current marital status, antenatal care (ANC) attendance, postnatal care (PNC) utilization, size of the child at birth, sex of the child, birth order, whether the child is a twin, media exposure, presence of skilled birth attendants, place of delivery, delivery by caesarean section, and maternal tetanus toxoid (TT) vaccination were included. Environmental and hygiene-related variables such as source of drinking water and type of toilet facility were also incorporated in the model^19–26^.

### Machine learning pipeline

**Fig. 1:**
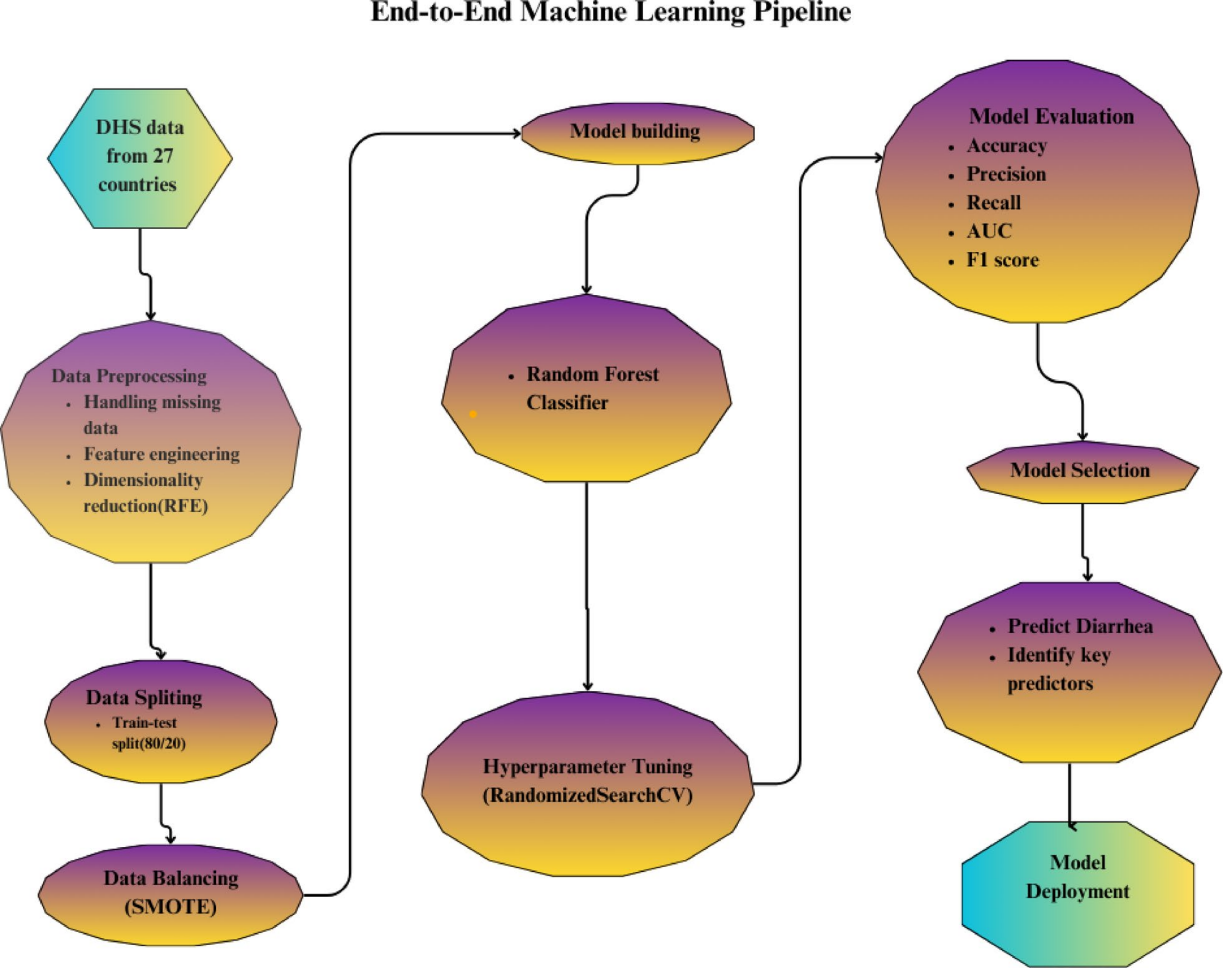
Conceptual diagram of the end-to-end machine learning pipeline.

The study implemented a structured machine learning pipeline comprising five critical phases. Data preprocessing was conducted first, where raw datasets underwent cleaning procedures including missing value imputation, categorical variable encoding, and feature normalization to ensure data quality. Subsequently, feature engineering was performed to select and transform the most predictive variables while maintaining model interpretability. The model development phase involved systematic algorithm selection and training, incorporating cross-validation techniques to optimize performance. Rigorous evaluation followed, utilizing standard metrics including precision, recall, and F1 scores to validate model effectiveness. Finally, the validated models were operationalized through API deployment on scalable platforms, enabling real-time predictive capabilities. This comprehensive methodological approach ensured reproducibility and practical applicability throughout the machine learning lifecycle (Fig. 1).

### Data processing

To address missing values, Multiple Imputation by Chained Equations (MICE) was applied, which iteratively models each variable with missing data using other variables in the dataset. Categorical variables were encoded via one-hot encoding, while numerical features were standardized using Z-score normalization. To systematically reduce dimensionality and enhance model performance, mutual information score was used for feature selection, and variables such as child is a twin, smoking cigarettes, delivery by cesarean section, age at first birth, wanted last child, permission to seek medical help, type of toilet facility, postnatal care (PNC), husband’s education, and not wanting to go to a health facility were removed from the analysis due to scores less than one. Following feature selection, correlation analysis was conducted to assess multicollinearity among the retained variables, supporting the stability and reliability of downstream machine learning models. Parity and Birth order was removed due to high correlation with No of living children, as it showed lower importance based on mutual information score. Additionally, scaling techniques was applied where necessary to ensure consistency in data representation (Table 1).

**Table 1:** List of independent variables considered for predicting diarrhea among under-five children, their data type, preprocessing transformations, and inclusion in the machine learning model.

Variable	Type	Preprocessing/transformation	Included in model?	Reason
Age of mother	Numerical	Z-score normalization	Yes	Retained, informative
Place of residence	Categorical	One-hot encoding	Yes	Retained, informative
Highest educational level	Categorical	One-hot encoding	Yes	Retained, informative
Wealth index	Categorical	One-hot encoding	Yes	Retained, informative
Maternal literacy	Categorical	One-hot encoding	Yes	Retained, informative
Smoking status (smokes cigarettes)	Categorical	One-hot encoding	No	Low mutual information score
Husband’s education level	Categorical	One-hot encoding	No	Low mutual information score
Current employment status	Categorical	One-hot encoding	Yes	Retained, informative
Number of living children	Numerical	Z-score normalization	Yes	Retained, informative
Parity	Numerical	Z-score normalization	No	High correlation with number of living children
Age at first birth	Numerical	Z-score normalization	No	Low mutual information score
Last child wanted	Categorical	One-hot encoding	No	Low mutual information score
Fertility preference	Categorical	One-hot encoding	Yes	Retained, informative
Permission to seek medical help	Categorical	One-hot encoding	No	Low mutual information score
Money for treatment difficulty	Categorical	One-hot encoding	Yes	Retained, informative
Distance to health facility	Numerical	Z-score normalization	Yes	Retained, informative
Reluctance to go alone	Categorical	One-hot encoding	No	Low mutual information score
Marital status	Categorical	One-hot encoding	Yes	Retained, informative
ANC attendance	Categorical	One-hot encoding	Yes	Retained, informative
PNC utilization	Categorical	One-hot encoding	No	Low mutual information score
Size of child at birth	Categorical	One-hot encoding	Yes	Retained, informative
Sex of child	Categorical	One-hot encoding	Yes	Retained, informative
Birth order	Numerical	Z-score normalization	No	High correlation with number of living children
Child is a twin	Categorical	One-hot encoding	No	Low mutual information score
Media exposure	Categorical	One-hot encoding	Yes	Retained, informative
Skilled birth attendant	Categorical	One-hot encoding	Yes	Retained, informative
Place of delivery	Categorical	One-hot encoding	Yes	Retained, informative
Delivery by cesarean	Categorical	One-hot encoding	No	Low mutual information score
Maternal TT vaccination	Categorical	One-hot encoding	Yes	Retained, informative
Source of drinking water	Categorical	One-hot encoding	Yes	Retained, informative
Type of toilet facility	Categorical	One-hot encoding	No	Low mutual information score

### Data balancing

Given the pronounced class imbalance (approximately 1:6) between children with diarrhea (minority class) and those without (majority class), we applied the Synthetic Minority Over-sampling Technique (SMOTE) to address potential bias toward the majority class. SMOTE generates synthetic minority-class samples by interpolating between existing minority observations in the feature space, rather than duplicating instances, thereby reducing the risk of overfitting while improving class separability. After applying SMOTE, the dataset achieved a balanced 1:1 class distribution, which is known to enhance classifier learning, stabilize decision boundaries, and improve performance metrics, particularly sensitivity and recall, for the minority class in imbalanced health datasets^27,28^.

### Model selection

Following rigorous data preprocessing, the Random Forest (RF) classifier was selected based on a systematic model selection strategy that jointly considered predictive performance and deployment feasibility. The choice was informed by prior evidence demonstrating that ensemble tree-based methods perform competitively across a wide range of structured health datasets when compared with support vector machines, boosting algorithms, and neural networks, particularly under moderate sample sizes and heterogeneous feature spaces^29^. The RF algorithm is particularly suitable for the characteristics of the study data, as it effectively models nonlinear relationships, accommodates high-dimensional feature spaces, and handles mixed data types without requiring strong parametric assumptions. Its ensemble learning mechanism reduces variance through aggregation of multiple decision trees, thereby improving generalization and mitigating overfitting relative to single-tree models and highly complex learners^30–34^. In addition, RF provides intrinsic feature importance measures, enabling transparent assessment of predictor contributions and supporting domain validation, an essential requirement in applied health and epidemiological research. Compared with deep neural networks and highly optimized gradient boosting models, RF achieves stable performance with lower computational overhead, fewer tuning requirements, and improved reproducibility, making it suitable for resource-constrained and scalable deployment settings^27,35,36^.

### Model training

After stratified splitting of the dataset into 80% training and 20% testing sets, the training data were balanced using SMOTE, ensuring equal representation of children with and without diarrhea. The Random Forest model was then trained on this balanced training set, allowing the algorithm to learn effectively from both classes without bias toward the majority class. The test set remained unmodified, preserving the original distribution, so that model evaluation reflected real-world performance.

### Hyperparameter tuning

Hyperparameter optimization for the Random Forest Classifier was performed using Randomized Search Cross-Validation (RandomizedSearchCV) from the scikit-learn library. This method was selected over exhaustive grid search due to its computational efficiency in exploring a wide hyperparameter space while maintaining a robust search strategy. The tuning process was conducted after applying SMOTE to the training data to address class imbalance, ensuring that the hyperparameter optimization occurred on a representative and balanced dataset. The search space was deliberately designed to cover both typical default ranges and extended values based on empirical evidence from similar healthcare prediction tasks^37–39^. The defined parameter distributions were: n_estimators (100–500), max_depth (None, and integer values from 5 to 25), min_samples_split (2, 5, 10), min_samples_leaf (1, 2, 4), max_features (‘sqrt’, ‘log2’, None), and bootstrap (True, False). A total of 50 random combinations were sampled from this space, and each candidate model was evaluated using 10-fold cross-validation on the training set. The primary optimization criterion was the F1-score, as it provides a balanced measure of precision and recall, which is critical in medical prediction contexts where both false positives and false negatives carry significant implications. The combination that yielded the highest mean cross-validation F1-score was automatically selected by RandomizedSearchCV as the optimal hyperparameter set. The final selected values were: n_estimators = 300, max_depth = 15, min_samples_split = 2, min_samples_leaf = 1, max_features=’sqrt’, and bootstrap = True (Table 2). These parameters were then used to train the final model, which was evaluated on the held-out test set using accuracy, precision, recall, F1-score, and AUC.

**Table 2:** Hyperparameter ranges explored using RandomizedSearchCV, the best-performing values selected for the Random Forest model.

Hyperparameter	Search range	Best value selected
n_estimators	100–500	300
max_depth	None, 5–25	15
min_samples_split	2, 5, 10	2
min_samples_leaf	1, 2, 4	1
max_features	‘sqrt’, ‘log2’, None	‘sqrt’
Bootstrap	True, False	True

### Model evaluation and performance metrics

In this analysis, we used a rigorous assessment framework to evaluate the performance of our machine learning models, ensuring reliability and effectiveness in real-world applications.

- **Accuracy**: Proportion of correct predictions among all predictions.
- **Precision**: Proportion of predicted positives that are actually positive (TP/(TP + FP)), reflecting prediction quality.
- **Recall (Sensitivity)**: Proportion of actual positives correctly identified (TP/(TP + FN)); high recall is critical where missing positives has serious consequences.
- **Area Under the ROC Curve (AUC)**: Measures the model’s ability to discriminate between positive and negative classes across all classification thresholds; higher values indicate better separation of classes regardless of class distribution.
- **F**_**1**_**-score**: Harmonic mean of precision and recall, providing a balanced measure, particularly important when both false positives and false negatives matter.

### Model deployment with flask

**Fig. 2:**
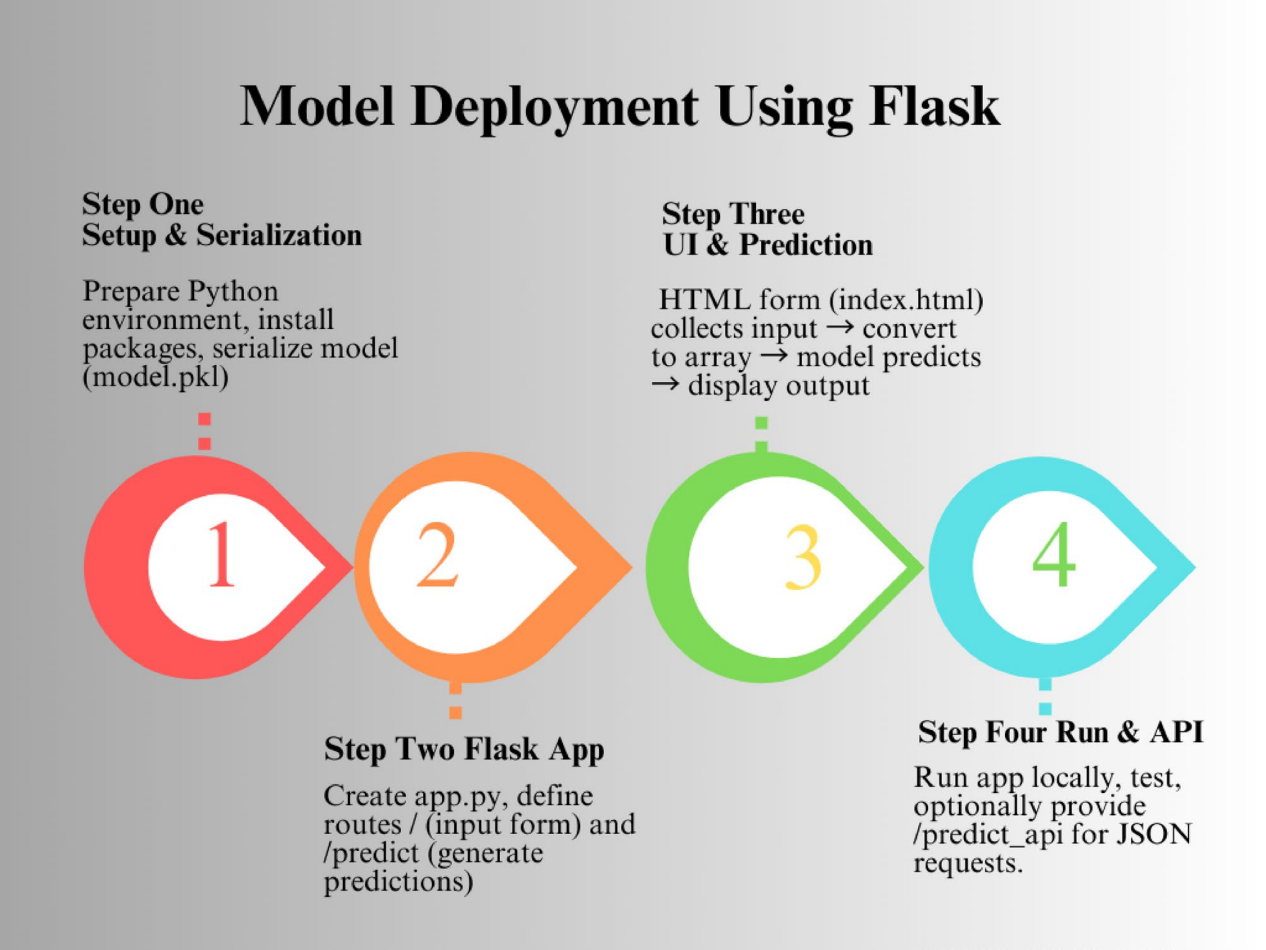
Machine learning model deployment with flask: a step-by-step guide from training to scalable API integration.

In this study, the trained Random Forest classifier was deployed as the final step, transforming it from a validated research model into an operational decision-support tool within a health information system. The process involved optimizing the model, serializing it into a portable format, and preparing the production environment to enable practical, real-world predictions for public health interventions. A web-based Application Programming Interface (API) was then developed using the Flask framework, which exposed the model’s functionality as an accessible service. In this architecture, incoming HTTP requests containing new input data were routed through the API, processed by the Random Forest model, and returned to the client application in a standardized format, typically JSON. This approach abstracted the internal complexities of the machine learning pipeline, allowing seamless integration with diverse client systems such as health information platforms, web applications, or mobile health tools. Furthermore, the deployment design considered essential aspects of scalability, security, interoperability, and performance monitoring to ensure that the model could operate reliably and sustainably in real-world public health environments (Fig. 2).

### Flask for ML model serving

Flask is a lightweight and flexible Python web framework that is well-suited for serving machine learning models as web services or APIs. Its minimalist design enables the creation of robust and scalable endpoints while focusing on the essential functionality of model serving. For development, the process typically begins with creating a dedicated virtual environment (e.g., using python3 -m venv flaskenv) to isolate dependencies and avoid conflicts with other projects, followed by activating the environment and installing Flask (pip install Flask). A basic Flask application is initialized with app = Flask(__name__), and routes are defined using the @app.route() decorator to specify URL paths and corresponding request-handling functions. For initial testing, a simple home or health check endpoint can be implemented, and running the application in debug mode (app.run(debug=True)) provides automatic server reloads and detailed error messages to facilitate troubleshooting. In the context of machine learning deployment, Flask functions as a lightweight API gateway, translating incoming HTTP requests from client applications into calls to the trained model, processing the model’s predictions, and returning standardized responses (typically in JSON format). This architecture abstracts the model’s internal operations, enabling seamless integration with diverse systems such as web platforms, mobile applications, and backend services, while supporting broader considerations for scalability, interoperability, and security.

### Building a RESTful API for predictions

**Fig. 3:**
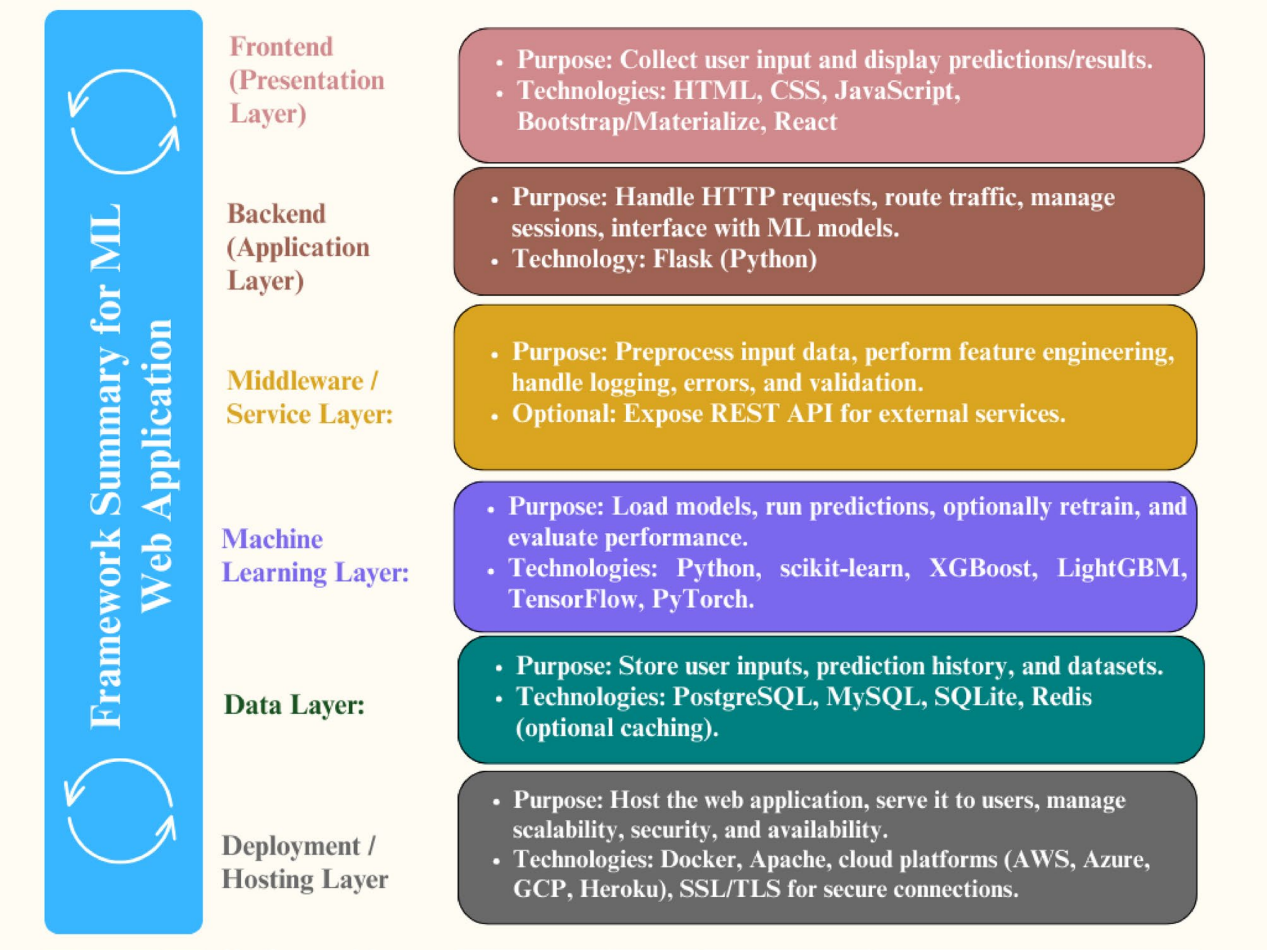
Framework for flask-based machine learning model deployment.

In this study, deploying the trained Random Forest classifier with Flask involved building a RESTful API capable of receiving input data, processing it through the model, and returning predictions. RESTful APIs are widely adopted in AI, machine learning, and data science for tasks such as data retrieval, manipulation, and prediction services. For securely and efficiently transmitting input data to the model, the HTTP POST method was employed, as it sends data in the body of the request rather than exposing it in the URL. The implementation included an app.py file containing the essential components for loading the model, defining the prediction endpoint, and handling incoming requests. For web-based interaction, an index.html file was placed in a templates directory to enable a form-driven interface. A critical element of this deployment was ensuring strict adherence to the model’s input contract, which defines the exact feature order, data types, format, and preprocessing transformations expected by the model. Incoming data, whether received via a web form, JSON payload, or other source, was validated and transformed to match the preprocessing pipeline used during training, including scaling, encoding, feature engineering, and imputation. Maintaining this consistency was essential to prevent inaccurate predictions or runtime errors, making the replication of preprocessing logic a non-negotiable requirement for reliable, real-time inference in the production environment (Fig. 3).

### Conversational AI integration as an informational support

To enhance interpretability and user engagement without altering predictive inference, a conversational artificial intelligence (AI) chatbot interface was integrated into the web-based application as an informational support (https://www.chatbase.co). The predictive architecture remains unchanged: the Random Forest classifier serves as the sole predictive engine and single source of truth within the system. All diarrhea risk predictions are generated exclusively by the trained and internally validated Random Forest model deployed on the Flask backend. The conversational interface does not perform prediction, does not access model weights, does not modify risk scores, and does not override classification decisions. It operates independently at the user interface level and has no programmatic interaction with the backend prediction pipeline. No bidirectional integration exists between the large language model (LLM) service and the serialized machine learning model. Consequently, predictive outputs remain deterministic and fully reproducible given identical inputs.

**Fig. 4:**
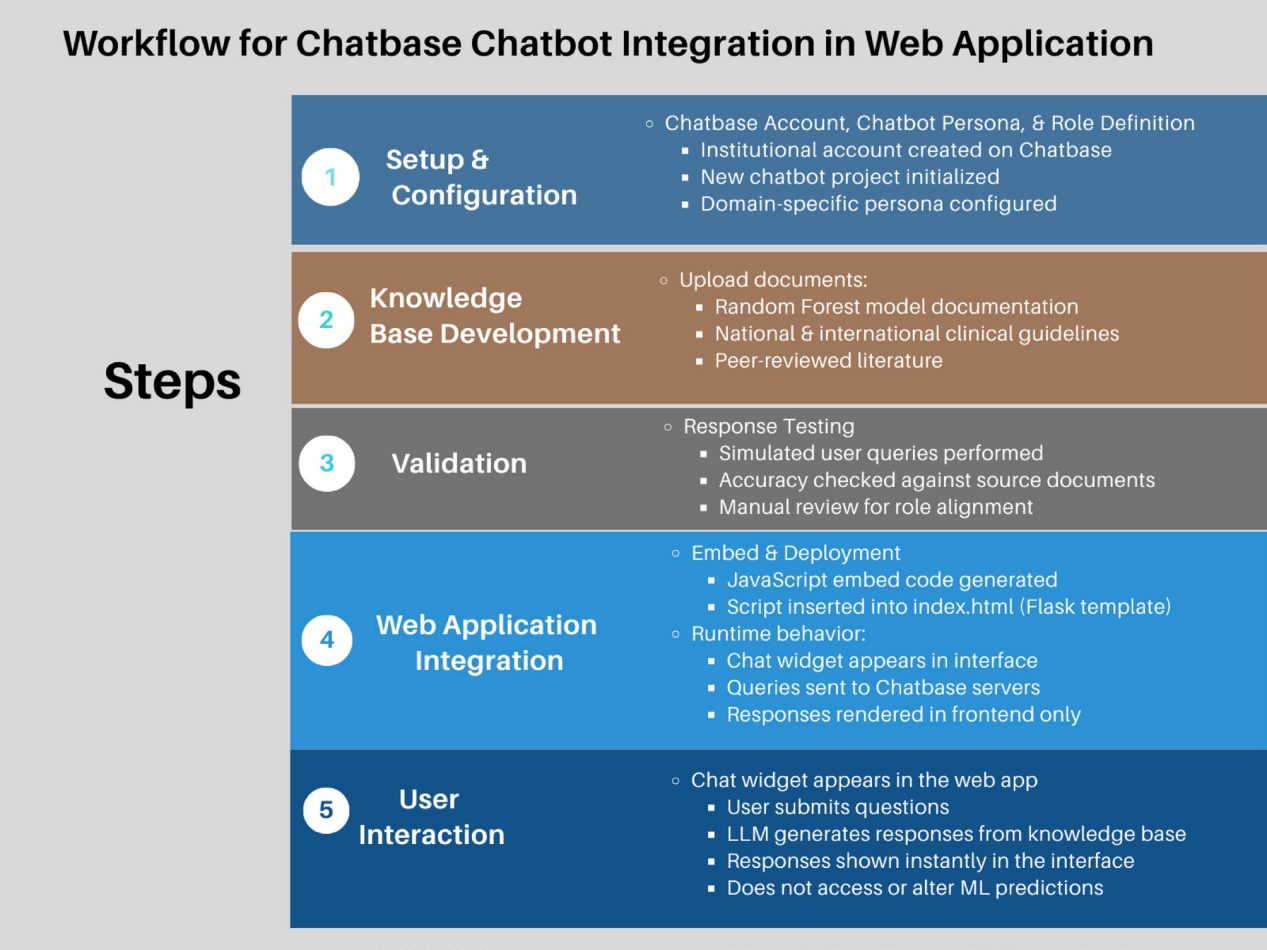
Workflow showing the integration of a chatbase AI chatbot into the web application, with setup, knowledge base development, LLM validation, embed deployment, and user interaction, operating independently of the Random Forest prediction engine.

The chatbot was developed following a structured and reproducible workflow. An institutional Chatbase account was created, and a dedicated chatbot instance was configured with a domain-specific persona (“Diarrhea Prediction Assistant”). System-level instructions were defined to restrict the chatbot’s role to explaining model input variables, clarifying prediction outputs, and providing general evidence-based educational information on childhood diarrhea management. The knowledge base was constructed by uploading: (1) detailed documentation of the Random Forest model (including features, preprocessing procedures, and feature importance rankings), (2) relevant national and international clinical guidelines, and (3) peer-reviewed literature on diarrheal disease epidemiology and interventions in Sub-Saharan Africa. Behavioral and safety parameters were configured to ensure clinically neutral responses, prevent speculative diagnostic statements, and avoid predictive functionality. The chatbot was subsequently tested using simulated user queries to confirm alignment with source documents and to verify that it neither accessed patient-level data nor interacted with the predictive algorithm. Following validation, the unique JavaScript embed code generated by Chatbase was inserted into the HTML template (index.html) of the Flask-based web application. Within this system architecture, the chatbot operates as a client-side interactive widget independent of the backend model. User queries are processed on Chatbase’s servers against the indexed knowledge base, and responses are rendered directly in the frontend interface without accessing or modifying the machine learning pipeline (Fig. 4).

### Model integrity

The Random Forest classifier remains the sole prediction engine in the system. The model was serialized after training, and its preprocessing pipeline (encoding, scaling, feature schema, and feature order) was preserved as a fixed artifact. This ensures that inference during deployment strictly replicates the training environment, thereby maintaining reproducibility.The conversational AI assistant operates independently at the user interface level and has no programmatic access to the serialized model object, prediction thresholds, or feature transformation logic. This architectural separation ensures that predictive inference remains transparent, reproducible, and auditable. The machine learning model therefore remains the exclusive source of predictive truth within the system.

## Results

**Fig. 5:**
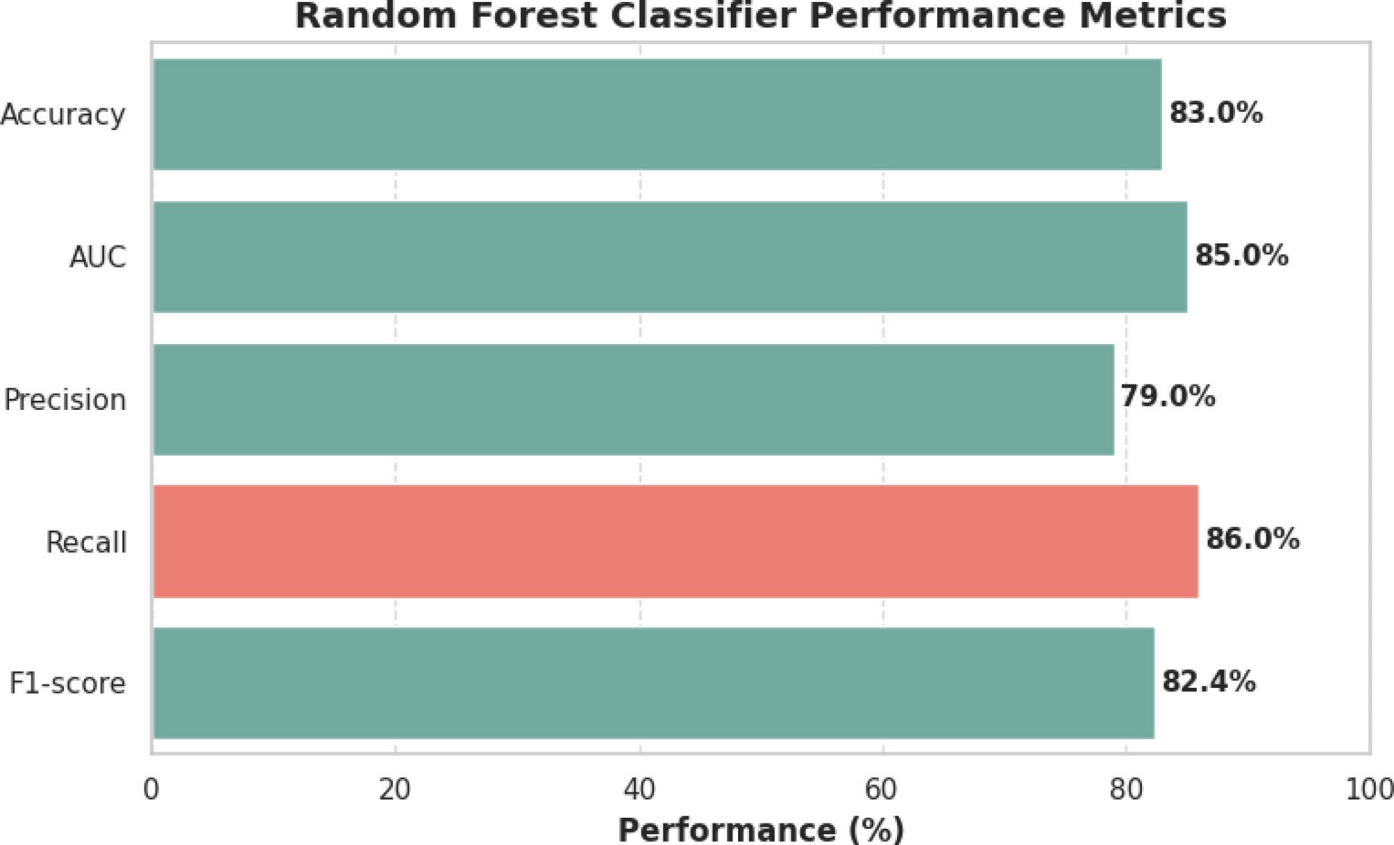
Performance metrics of the Random Forest classifier for identifying children under five with diarrhea across selected Sub-Saharan African countries.

The Random Forest classifier demonstrated strong predictive performance in identifying children under five with diarrhea, achieving an overall accuracy of 83% and an AUC of 85%, indicating excellent discrimination between children with and without diarrhea. The model’s precision (79%) and recall (86%), reflected in an F1-score of 82.4%, highlight its ability to balance correctly identifying true cases while minimizing false positives. These results can be attributed to several factors in the modeling process: hyperparameter tuning via RandomizedSearchCV optimized the number of trees, tree depth, and feature splits for best performance; SMOTE-based balancing of the training dataset ensured that the model effectively learned from the minority class without bias; and feature engineering and dimensionality reduction helped reduce noise and multicollinearity, allowing the model to focus on the most informative predictors. Overall, the combination of these strategies contributed to the observed high performance and reliability of the model in detecting diarrhea cases across the study population (Fig. 5).

### Flask-based ML web application with chatbot

An interactive web application was developed to translate the predictive model into a practical decision-support tool for diarrhea risk assessment among children under five in Sub-Saharan Africa. The application is powered by a Random Forest classifier trained on nationally representative DHS data, achieving a predictive accuracy of 79.6% and a recall of 84.1%, ensuring high sensitivity in identifying high-risk cases. The platform incorporates the most influential determinants identified during model development, including maternal and household characteristics (e.g., mother’s age, educational attainment, household wealth index, and access to improved water sources), healthcare access indicators (e.g., antenatal care attendance, tetanus toxoid immunization status, and distance to health facilities), and behavioral attributes (e.g., media exposure and financial barriers to treatment). Users can enter these variables through an intuitive interface with dropdown menus and checkboxes, enabling efficient data capture. Upon submission, the system generates individualized risk predictions with clear risk stratification to guide targeted interventions.

**Fig. 6:**
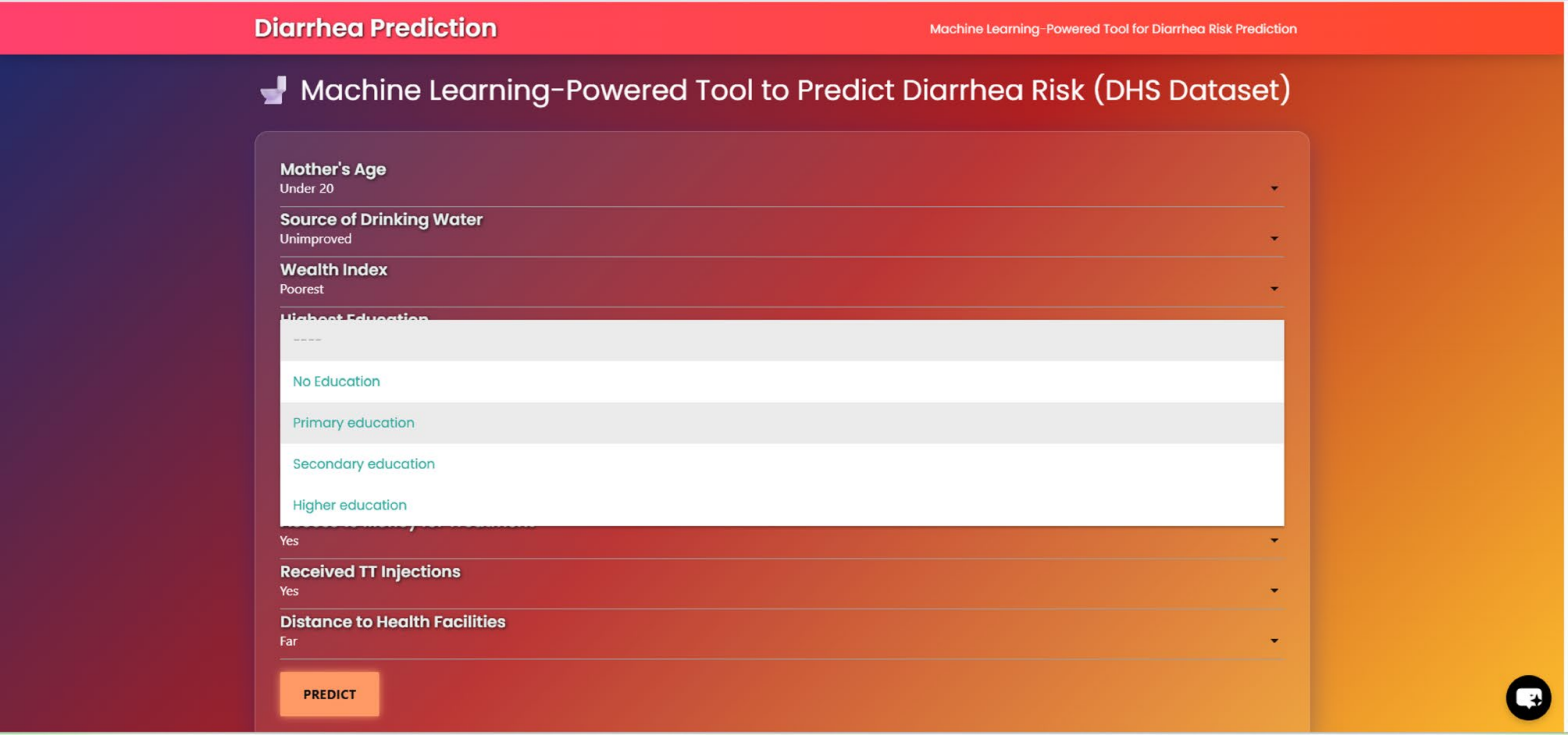
User interface 2 for machine learning-based diarrhea risk prediction tool (DHS dataset).

To enhance user engagement and knowledge dissemination, a chatbot feature was integrated at the bottom of the web page. This chatbot is trained on the lates research findings t and relevant childhood diarrhea management guidelines, allowing users to easily access context-specific information, clarification on risk outputs, and educational content related to prevention and treatment. The chatbot successfully fields queries related to the prediction inputs, explains the public health significance of key factors identified by the model (e.g., water source, maternal education), and provides guideline-based information on prevention and management. This integration ensures that the model’s output is not merely a numerical score but is contextualized within an interactive AI-assisted framework that supports understanding, decision-making, and knowledge dissemination, fulfilling the goal of a practical, assistive tool for end-users in public health (Fig. 6).

## Discussion

Random Forest model, optimized via RandomizedSearchCV and trained on SMOTE-balanced data from 289,720 children across 27 SSA countries, demonstrated strong and balanced predictive performance for identifying diarrhea in children under five. The predictive performance of the present study is broadly comparable with, and in some aspects superior to, previously published machine learning studies on childhood diarrhea using DHS data. Our Random Forest model achieved an accuracy of 79.6%, recall of 84.1%, precision of 79%, F1-score of 82.4%, and an AUC of 85%, demonstrating strong discriminatory power and high sensitivity for identifying true diarrhea cases. Compared with a multi-classifier DHS-based study that reported higher accuracy AUC 72% for logistic regression, our model achieved a higher recall 84.1%, indicating improved detection of positive cases, which is critical for a life-threatening condition^40^. Relative to single-country studies, our results substantially outperformed findings from Uganda, where gradient boosting achieved an accuracy of approximately 70%^41^, likely due to the limited dataset size and reduced population heterogeneity. In contrast, our accuracy was slightly lower than that reported in a Zimbabwean study using logistic regression (85%)^42^; however, logistic regression models are known to be less effective in handling complex nonlinear relationships and class imbalance, whereas our approach prioritized sensitivity and generalizability through SMOTE-based balancing and ensemble learning across multiple countries^43^.

Beyond predictive accuracy, a major contribution of this study lies in its focus on real-world deployment, an aspect that remains underrepresented in the existing diarrhea prediction literature. While most published machine learning studies in child health report performance metrics only, few provide reproducible deployment frameworks suitable for integration into health information platforms. Systematic reviews of machine learning in healthcare consistently highlight this translational gap, noting that a large proportion of models never progress beyond experimental environments^44^. By deploying the optimized Random Forest model as a Flask-based RESTful API and integrating it into an interactive web application, the present study aligns with emerging best practices and demonstrates a complete data-to-deployment pipeline^45^. This end-to-end approach enhances the practical relevance of the model and supports its use as a scalable decision-support tool in resource-limited public health settings. Consequently, this work contributes not only robust multicounty predictive evidence but also a tangible implementation framework, thereby strengthening its positioning within the current machine learning and digital health literature.

### Strengths and limitations

A key strength of this study is that it addresses a largely untracked gap in the literature by going beyond model development and performance reporting to demonstrate practical model deployment. Unlike many machine learning studies that remain confined to experimental environments, this work presents an end-to-end pipeline, operationalizing the predictive model as a deployable API and interactive application. This contributes methodological and practical evidence on how machine learning models can be translated into usable decision-support tools, thereby enhancing reproducibility and real-world relevance.:. Although the model was deployed as a standalone Flask-based application, its semantic and conceptual interoperability with existing health information systems has not been formally evaluated. While the API supports structured data exchange, integration into national platforms (e.g., DHIS2 or electronic health records) requires alignment with standardized data models and coding systems, and its ability to map clinical concepts using frameworks such as HL7 FHIR, LOINC, and SNOMED CT remains untested. Additionally, because the model inputs were derived from DHS variable structures, discrepancies between survey-based variables and routine facility data may necessitate further mapping and harmonization prior to real-world implementation. Finally, the system has not yet been evaluated in actual public health practice, leaving questions regarding usability, workflow integration, and user acceptance unresolved. These limitations underscore the need for future research focused on interoperability validation, continuous model monitoring and drift detection, and real-world implementation studies to assess system performance and practical utility in authentic public health settings.

## Conclusion

This study successfully developed and deployed a machine learning model to predict diarrhea among children under five in Sub-Saharan Africa, leveraging DHS)data from 27 countries. The end-to-end pipeline encompassed rigorous data preprocessing, feature engineering, model selection (Random Forest), hyperparameter tuning, and evaluation, achieving balanced performance metrics (accuracy: 79.6%, F1-score: 80.7%, recall: 84.1%). The deployment phase utilized Flask to operationalize the model as a RESTful API, ensuring scalability, interoperability, and real-time usability in public health settings. By bridging the gap between theoretical research and practical implementation, this work contributes to the growing field of ML operations, demonstrating how machine learning solutions can transition from experimental environments to actionable tools for healthcare decision-making.

### Recommendation

To translate this proof-of-concept into public health impact, it is recommended to pilot the tool in high-burden regions with community health workers to evaluate usability, workflow integration, and the effectiveness of linking predictions to interventions like ORS distribution. This must be paired with formal partnerships with regional health ministries to assess semantic interoperability and securely integrate the model with existing national health information systems. To ensure sustained safety and performance, automated systems for drift detection should be implemented, supported by quarterly model retraining with updated data. Finally, developing training and feedback mechanisms will be essential to help frontline staff interpret model outputs and embed them into routine clinical workflows effectively.

## Data Availability

The datasets analyzed in the current study are available in the public domain through the Measure DHS website (http://www.measuredhs.com).

## Declarations

### Patient and public involvement

No patients or members of the public were directly involved in the design, conduct, reporting, or dissemination of this research, as it utilized secondary, de-identified DHS datasets.
